# Osteopetrosis

**DOI:** 10.1186/1750-1172-4-5

**Published:** 2009-02-20

**Authors:** Zornitza Stark, Ravi Savarirayan

**Affiliations:** 1 Genetic Health Services Victoria, and Murdoch Childrens Research Institute, Melbourne, Australia; 2 Department of Paediatrics, University of Melbourne, Melbourne, Australia

## Abstract

Osteopetrosis ("marble bone disease") is a descriptive term that refers to a group of rare, heritable disorders of the skeleton characterized by increased bone density on radiographs. The overall incidence of these conditions is difficult to estimate but autosomal recessive osteopetrosis (ARO) has an incidence of 1 in 250,000 births, and autosomal dominant osteopetrosis (ADO) has an incidence of 1 in 20,000 births. Osteopetrotic conditions vary greatly in their presentation and severity, ranging from neonatal onset with life-threatening complications such as bone marrow failure (*e.g*. classic or "malignant" ARO), to the incidental finding of osteopetrosis on radiographs (*e.g*. osteopoikilosis). Classic ARO is characterised by fractures, short stature, compressive neuropathies, hypocalcaemia with attendant tetanic seizures, and life-threatening pancytopaenia. The presence of primary neurodegeneration, mental retardation, skin and immune system involvement, or renal tubular acidosis may point to rarer osteopetrosis variants, whereas onset of primarily skeletal manifestations such as fractures and osteomyelitis in late childhood or adolescence is typical of ADO. Osteopetrosis is caused by failure of osteoclast development or function and mutations in at least 10 genes have been identified as causative in humans, accounting for 70% of all cases. These conditions can be inherited as autosomal recessive, dominant or X-linked traits with the most severe forms being autosomal recessive. Diagnosis is largely based on clinical and radiographic evaluation, confirmed by gene testing where applicable, and paves the way to understanding natural history, specific treatment where available, counselling regarding recurrence risks, and prenatal diagnosis in severe forms. Treatment of osteopetrotic conditions is largely symptomatic, although haematopoietic stem cell transplantation is employed for the most severe forms associated with bone marrow failure and currently offers the best chance of longer-term survival in this group. The severe infantile forms of osteopetrosis are associated with diminished life expectancy, with most untreated children dying in the first decade as a complication of bone marrow suppression. Life expectancy in the adult onset forms is normal. It is anticipated that further understanding of the molecular pathogenesis of these conditions will reveal new targets for pharmacotherapy.

## Disease name and synonyms

The term osteopetrosis is derived from the Greek 'osteo' meaning bone and 'petros', stone. Osteopetrosis is variably referred to as 'marble bone disease' and 'Albers-Schönberg disease', after the German radiologist credited with the first description of the condition in 1904 [1].

## Definition and classification

Osteopetrosis comprises a clinically and genetically heterogeneous group of conditions that share the hallmark of increased bone density on radiographs. The increase in bone density results from abnormalities in osteoclast differentiation or function. The Nosology Group of the International Skeletal Dysplasia Society classifies increased bone density conditions into several distinct entities based on clinical features, mode of inheritance and underlying molecular and pathogenetic mechanisms (Table 1) [2].

**Table 1:** Classification of osteopetrotic conditions, modified from the Nosology and Classification of Genetic Skeletal disorders (2006 revision)[2]

**Condition**	**Inheritance**	**OMIM No**	**Gene**	**Mutation mechanism**	**Protein**	**Rodent model**
Osteopetrosis, severe neonatal or infantile forms	AR	259700	*TCIRG1*	Loss of function	Subunit of V-ATPase pump	*Oc/oc* *Tcirg1-/-*
			
	AR		*CLCN7*	Loss of function	Chloride channel	*Clcn7-/-*
			
	AR		*OSTM1*	Loss of function	Osteopetrosis associated transmembrane protein	*Gl/gl*
			
	AR		*RANKL*	Loss of function	Receptor Activator for Nuclear Factor κ B Ligand	*Tnfsfl 1-/-*
			
	AR		*RANK*	Loss of function	Receptor Activator for Nuclear Factor κ B	*Tnfrsf11a-/-*

Osteopetrosis, intermediate form	AR	259710	*CLCN7*		Chloride channel	
			
	AR		*PLEKHM1*	Loss of function	Pleckstrin homology domain containing family M, member 1	*ia *rat

Osteopetrosis with renal tubular acidosis	AR	259730	*CAII*	Loss of function	Carbonic anhydrase II	

Osteopetrosis, late-onset form ('Albers-Schönberg disease')	AD	166600	*CLCN7*	Dominant negative	Chloride channel	

Osteopetrosis with ectodermal dysplasia and immune defect (OLEDAID)	XL	300301	*IKBKG (NEMO)*	Loss of function	Inhibitor of kappa light polypeptide gene enhancer, kinase of	*Nemo-/-*

Leukocyte adhesion deficiency syndrome (LAD-III) and osteopetrosis	AR		*Kindlin-3*	Loss of function	Kindlin-3	*Kind3-/_*
			
	AR		*CalDAG-GEF1*	Loss of function	Calcium and diacylglycerol-regulated guanine nucleotide exchange factor 1	*CalDAG-GEF1-/-*

Pycnodysostosis	AR	265800	*CTSK*	Loss of function	Cathepsin K	*cathK-/-*

Osteopoikilosis	AD	155950	*LEMD3*	Loss of function	LEM domain-containing 3	

Melorheostosis with osteopoikilosis	AD	155950	*LEMD3*	Loss of function	LEM domain-containing 3	

Dysosteosclerosis	AR	224300				

Osteomesopyknosis	AD	166450				

Osteopathia striata congenita with cranial stenosis	XL	300373	*WTX*	Loss of function	Wilms tumour gene on the X chromosome	

Osteosclerosis, Stanescu type	AD	122900				

## Epidemiology

These conditions are rare, and their overall incidence is difficult to estimate. Autosomal recessive osteopetrosis has an incidence of 1 in 250,000 births, with a particularly high incidence reported in Costa Rica (3.4:100,000) [3]. Autosomal dominant osteopetrosis has an incidence of 5:100,000 births [4].

## Clinical descriptions

Osteopetrosis encompasses a group of highly heterogeneous conditions, ranging in severity from asymptomatic to fatal in infancy. The more severe forms tend to have autosomal recessive inheritance, while the mildest forms are observed in adults and are inherited in an autosomal dominant manner.

The increased bone mass can result in phenotypic features such as macrocephaly and altered craniofacial morphology, but more importantly impacts on other organs and tissues, notably the bone marrow and nervous systems. The key clinical manifestations, onset, severity, treatment, prognosis and recurrence risks for the main types of osteopetrosis are summarised in Table 2.

**Table 2:** Summary of the key clinical manifestations, onset, severity, treatment, prognosis and recurrence risks of the main types of osteopetrosis

**Osteopetrosis subtype**	**Autosomal recessive osteopetrosis (ARO)**			**X-linked osteopetrosis, lymphedema, anhidrotic ectodermal dysplasia and immunodeficiency (OLEDAID)**	**Intermediate osteopetrosis (IRO)**	**Autosomal dominant osteopetrosis** **(Albers-Schönberg** **disease)**
				
	**Classic**	**Neuropathic**	**ARO with RTA**			
**Genetic basis**	*TCIRG*	CLCN7, OSTM1	*Carbonic anhydrase II*	*IKBKG (NEMO)*	*CLCN7, PLEKHM1*	*CLCN7*

**Skeletal manifestations**			Increased bone density, diffuse and focal sclerosis of varying severity Modelling defects at metaphyses Pathological fractures Osteomyelitis Dental abnormalities: tooth eruption defects and dental caries						

**Other manifestations**	Pancytopaenia. Extramedullary haematopoiesis, hepatosplenomegaly. Cranial nerve compression (II, VII, VIII) Hydrocephalus Hypocalcaemia	As for classic ARO, but primary neurodegeneration, including retinal atrophy	Renal tubular acidosis. Developmental delay. Intracranial calcification. Cranial nerve compression. Bone marrow impairment rare.	Anhidrotic ectodermal dysplasia. Lymphedema. Immunodeficiency resulting in overwhelming infection.	Anaemia and extramedullary haematopoiesis Occasional optic nerve compression	Moderate haematological failure Cranial nerve compression

**Onset**	Perinatal	Perinatal	Infancy	Infancy	Childhood	Late childhood or adolescence

**Severity**	Severe	Severe	Moderate	Severe	Mild to moderate	Mild to moderate, occasionally severe

**Treatment**	Supportive HSCT	Supportive	Supportive May benefit from HSCT	Supportive	Supportive	Supportive

**Prognosis**	Poor Fatal in infancy	Poor Fatal in infancy	Variable	Poor Fatal in early childhood	Variable	Normal life expectancy

**Recurrence risk**	Parents of proband: 25% risk of recurrence in future pregnancies			If mother of proband carrier: 50% of male pregnancies affected	Parents of proband: 25% risk of recurrence in future pregnancies	50% in future pregnancies if one parent affected

*Autosomal recessive ("malignant") osteopetrosis (ARO) *is a life-threatening condition, which classically manifests in the first few months of life. The increase in bone density can paradoxically weaken the bone, resulting in a predisposition to fractures and osteomyelitis. The longitudinal growth of bones is impaired, resulting in short stature of varying degrees. Macrocephaly and frontal bossing develop within the first year, resulting in a typical facial appearance. The skull changes can result in choanal stenosis and hydrocephalus [5]. The expanding bone can narrow nerve foramina, resulting in blindness, deafness, and facial palsy. Hearing loss is estimated to affect 78% of individuals with ARO [6]. Tooth eruption defects and severe dental caries are also common. Children with ARO are at risk of developing hypocalcaemia, with attendant tetanic seizures and secondary hyperparathyroidism. The most severe complication of ARO is bone marrow suppression. The abnormal expansion of bone interferes with medullary haematopoiesis, resulting in life-threatening pancytopaenia, and secondary expansion of extramedullary haematopoiesis sites such as the liver and spleen.

*ARO variants*. It is important to differentiate classic ARO from some of the rarer variants. *Neuropathic ARO *is characterised by seizures in the setting of normal calcium levels, developmental delay, hypotonia, retinal atrophy with absent evoked visual potentials and sensorineural deafness [7]. It is due to primary neurodegeneration not dissimilar to neuronal ceroid-lipofuschinosis, a lysosomal storage disorder [8]. Reported brain MRI findings include significantly delayed myelinisation, diffuse progressive cortical and subcortical atrophy, and bilateral atrial subependymal heterotopias [7]. Electron microscopy of skin biopsies reveals swollen unmyelinated axons that contain spheroids, reduced numbers of myelinated axons and the presence of secondary lipofuscin-containing lysosomes in Schwann cells [9].

*ARO with renal tubular acidosis (RTA) *has a milder course where RTA and cerebral calcifications are typical [10]. Other clinical manifestations comprise an increased frequency of fractures, short stature, dental abnormalities, cranial nerve compression and developmental delay [11].

The presence of severe immunodeficiency with ectodermal changes is observed in *X-linked osteopetrosis, lymphedema, anhidrotic ectodermal dysplasia and immunodeficiency (OLEDAID*). Common variable immune deficiency (CVID) has been described in association with a particular subtype of osteoclast-poor ARO [12], and some patients with leukocyte adhesion deficiency syndrome (LAD-III) also suffer from severe osteopetrosis [13,14].

**Figure 1:**
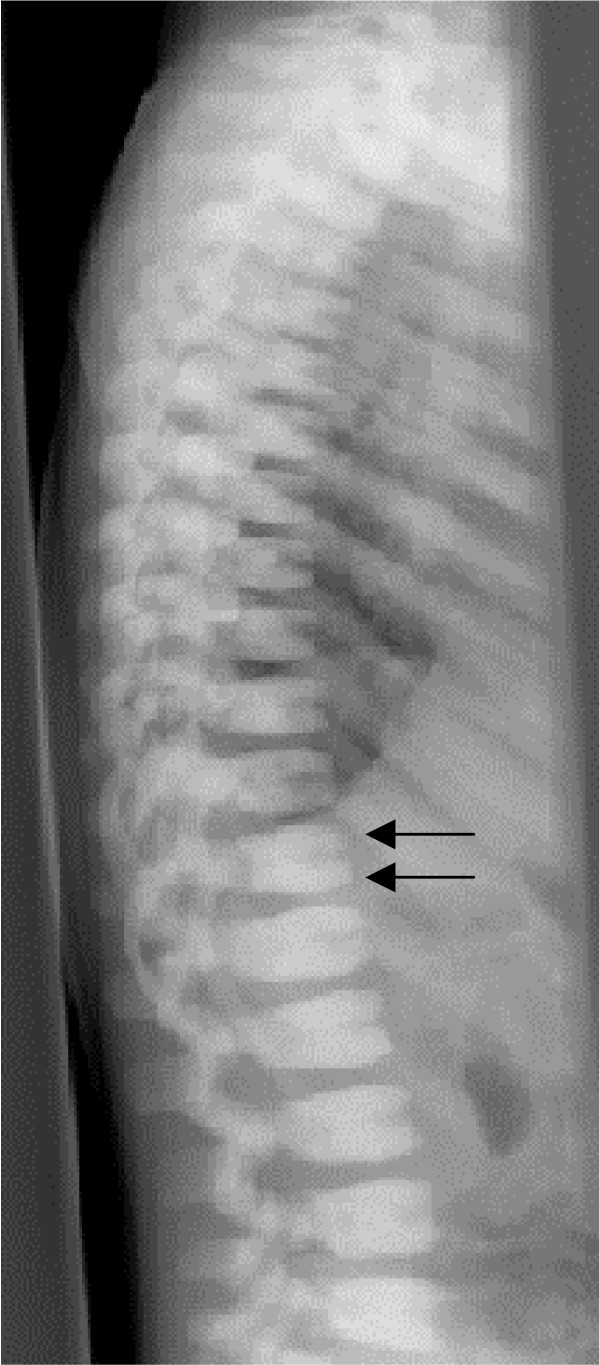
**ADO: lateral spine radiograph, age 4 years**. Note sclerosis of vertebral endplates (arrows) resulting in 'sandwich vertebrae' appearance.

*Autosomal dominant osteopetrosis (Albers-Schönberg disease) *typically has onset in late childhood or adolescence, and classically displays the radiographic sign of "sandwich vertebrae" (dense bands of sclerosis parallel to the vertebral endplates – Figure 1). The main complications are confined to the skeleton, including fractures, scoliosis, hip osteoarthritis and osteomyelitis, particularly affecting the mandible in association with dental abscess or caries [15]. Cranial nerve compression is a rare but important complication, with hearing and visual loss affecting around 5% of individuals.

**Figure 2:**
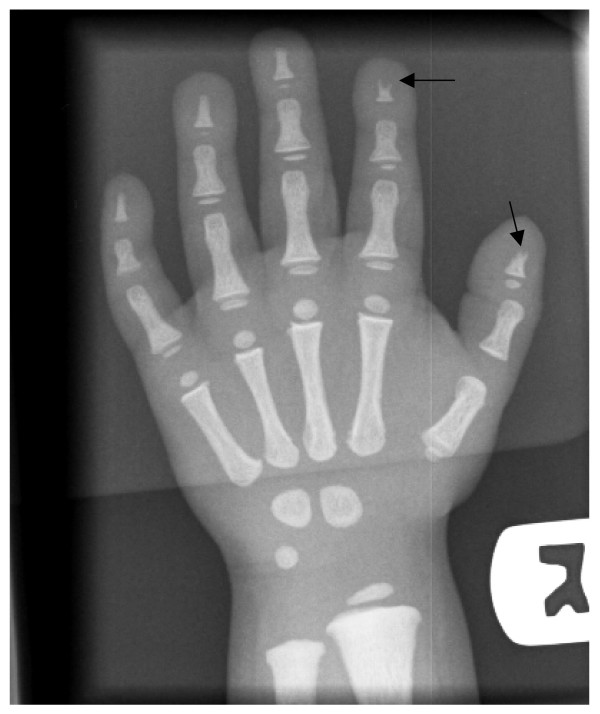
**Pycnodysostosis: Hand radiograph, age 3 years**. Note acro-osteolysis in the distal phalanx of thumb and index fingers (arrows) and generalised increased bone density.

**Figure 3:**
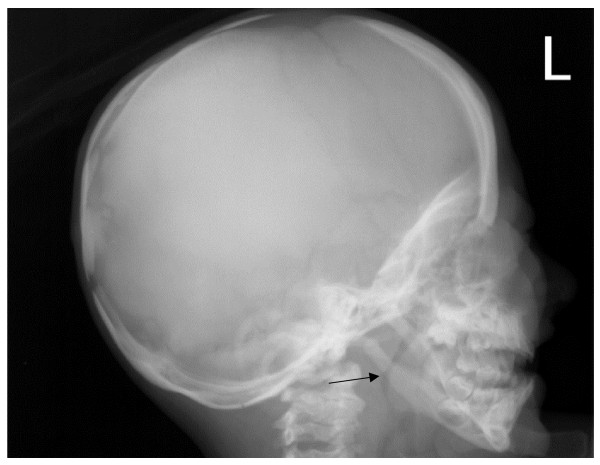
**Pycnodysostosis: Lateral skull radiograph, age 3 years**. Note loss of the mandibular angle (arrow) and increased thickness of vault.

*Pycnodysostosis *was first described by Maroteaux and Lamy in 1962 [16], and there is evidence that the French painter Henri de Toulouse-Lautrec [17] and the ancient Greek author Aesop [18] were afflicted by this condition. Pycnodysostosis is characterised by short stature, increased bone fragility, persistent open anterior fontanelle and acro-osteolysis of the terminal phalanges (Figure 2). The typical 'open mouth outline' facial appearance is due to frontal bossing, micrognatia, loss of the mandibular angle (Figure 3), and dental anomalies including persistence of deciduous teeth resulting a double row of teeth [19,20]. Other reported manifestations include joint hypermobility, obliteration of frontal and other sinuses [21], pituitary hypoplasia, cerebral demyelination [22], and hepatosplenomegaly [23].

*Dysosteosclerosis *was first described as a separate condition in 1968 [24] and is marked by the presence of skin changes (red-violet spots in a patchy distribution), developmental regression and an overall poor prognosis. It manifests in infancy and can be distinguished from other osteopetrotic conditions by platyspondyly, bowing of the long bones, and the lack of bone marrow involvement or acro-osteolysis [25]. Typically, the expanded areas of bone are relatively radiolucent, in contrast to the sclerosis of expanded areas seen in ARO [25].

*Osteopoikilosis *is a benign, usually asymptomatic condition diagnosed radiographically by the presence of multiple symmetrical circular/ovoid sclerotic opacities of the ischiae, pubic bones and the epimetaphysial regions of the short tubular bones. Osteopoikilosis can occur in isolation, or in association with elastic or collagen connective tissue naevi of the skin, a condition termed *Buschke-Ollendorff syndrome (BOS)*. Both osteopoikilosis and BOS are inherited in an autosomal dominant pattern. In some families and individuals, osteopoikilosis can also occur in association with *melorheostosis *[26-29]. Melorheostosis is usually a sporadic sclerosing bone condition manifesting in a sclerotomal distribution, frequently affecting one limb. There is cortical hyperostosis with thickening, which resembles dripping candle wax on radiographs. Melorheostosis can be asymptomatic or if severe can result in pain, stiffness, leg-length discrepancy and deformity.

*Osteopathia striata (OS) *occurs in isolation or with *cranial sclerosis (OS-CS)*. The key feature is longitudinal striation of the metaphyses of the long bones [30]. OS-CS in particular is a clinically heterogeneous condition, ranging from mild skeletal manifestations to multisystem organ involvement even within the same family [31]. The typical clinical features include macrocephaly, cleft palate and hearing loss; additional features including cardiac malformations, developmental delay, cranial nerve palsies, anal malformations, cataracts and nervous system malformations have been reported.

## Aetiology

**Figure 4:**
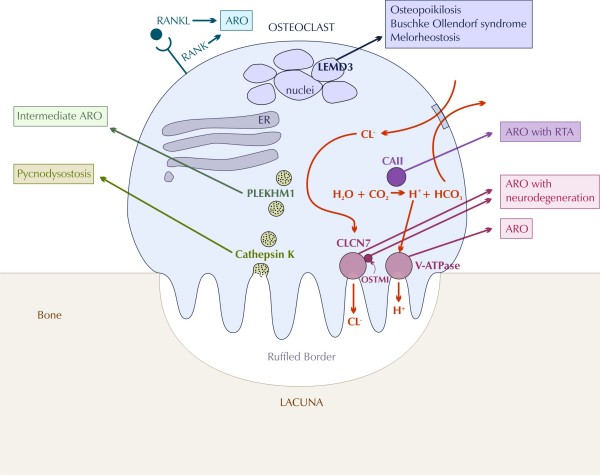
**Current model of the pathogenesis of osteopetrotic conditions in relation to normal osteoclast function, modified from Del Fattore et al **[77]** (ER: endoplasmic reticulum, ARO: autosomal recessive osteopetrosis, RTA: renal tubular acidosis)**.

Osteopetrosis is caused by failure of osteoclast differentiation or function and mutations in at least 10 genes have been identified as causative in humans (Table 1). The pathogenesis of osteopetrosis is best understood with reference to normal osteoclast development and function (Figure 4).

Osteoclasts are highly specialised cells, which degrade bone mineral and organic bone matrix. These processes are crucial for bone remodelling and the maintenance of bone biomechanical stability and mineral homeostasis. It is estimated that the adult skeleton is completely regenerated every 10 years [32]. Osteoclasts are derived from the mononuclear precursors in the myeloid lineage of haematopoietic cells that also give rise to macrophages [33]. The osteoclast precursors fuse, resulting in osteoclasts, which typically have 5–8 nuclei. By contrast, osteoblasts are derived from multipotent mesenchymal stem cells, which also give rise to chondrocytes, adipocytes and muscle cells.

In light of the common origin of osteoclasts and cells of the haematopoietic system, it is not surprising that mutations in molecules such as IKBKG(NEMO) [34] and more recently CalDAG-GEF1 [13] and kindlin-3 [14] have been implicated in the pathogenesis of ARO variants associated with immune system dysfunction. Other important signals for osteoclast differentiation include the ligand of receptor activator of nuclear factor-kappa B (RANKL) and M-CSF. *Op/op *mice which do not express functional M-CSF lack osteoclasts and have osteopetrosis [35], however human patients with osteopetrosis secondary to M-CSF deficiency are yet to be identified. Recently a kindred with *RANKL *gene mutation [36], and seven families with *RANK *gene mutation [12] and osteopetrosis have been described. Failure of osteoclast differentiation as a result of mutations in these genes accounts for the rare osteoclast-poor forms of ARO, in which no mature osteoclasts are present.

A fully differentiated osteoclast dissolves bone mineral and degrades bone matrix using specialised enzymes. Crucial to this function is cell polarisation, and in particular the formation of the ruffled border and sealing zone. These form the resorption lacuna where hydrochloric acid is actively secreted resulting in the dissolution of bone mineral hydroxyapatite.

Most forms of osteoclast-rich osteopetrosis are caused by defects in gene products involved in the acidification machinery. Acid secretion is dependent on two key molecules, which facilitate proton transport: the proton pump vacuolar ATPase (V-ATPase) and the chloride-specific ion channel, chloride channel 7 (CLCN-7) [37]. Homozygous mutations in the genes encoding the a3 subunit of V-ATPase (*TCIRG1*) and the CLCN-7 produce severe malignant osteopetrosis phenotypes in both humans and mice [37-40]. *TCIRG1 *mutations are responsible for autosomal recessive osteopetrosis in more than 50% of affected individuals [38,41] underscoring the crucial role of V-ATPase in osteoclast function. CLCN-7 on the other hand plays a key role in lysosomal acidification, which explains the severe neuronal storage and neurodegeneration in the CNS and retina in Clcn7-/- mice and in a subset of human ARO patients [8,42]. Dominant-negative mutations of CLCN-7 have been shown to cause ADO [43]. CLCN-7 is closely associated with another membrane protein, OSTM1 [44]. Mutations in the *OSTM1 *gene are found in grey-lethal mice and a subset of ARO patients with neurological involvement [45,46].

The protons and chloride ions that are expended in the acidification process need to be replenished intracellularly in order to avoid alkanization. This is achieved by carbonic anhydrase type II (CAII) and an anion exchanger. Given the key role of CAII in kidney function, it is not surprising that mutations in CAII result in ARO with tubular acidosis [47].

The collagen bone matrix is dissolved by two groups of enzymes, the matrix metalloproteinases (MMPs) and lysosomal cathepsins. Cathepsin K in particular has been identified as a key enzyme. It is secreted in the resorption lacuna [48,49] where it degrades collagen I at acidic pH. Inhibition of cathepsin K prevents matrix degradation [50,51], and deletion of the *cathepsin K *gene in mice leads to osteopetrosis [52,53]. Homozygous mutations in the human *cathepsin K *gene lead to pycnodysostosis [54,55].

The formation and maintenance of the osteoclast polarised membrane domains requires complex vesicular trafficking mechanisms and continuous remodelling of the osteoclast cytoskeleton. One protein, which plays a critical function in vesicle trafficking and acidification is PLEKHM1, and heterozygous mutations in this have been associated with intermediate forms of osteopetrosis [56,57].

Other signalling pathways are likely to be important in osteoclast function, and mutations in the *LEMD3 *gene which codes for an integral protein of the inner nuclear membrane thought to be involved in BMP and TGFβ signalling, result in osteopoikilosis, Buschke-Ollendorff syndrome and melorheostosis [58,59]. WNT-related signalling defects (of *PORCN *[60,61] and *WTX *[62] genes) have recently been associated with hyperostotic phenotypes (osteopathia striata in Goltz syndrome and Osteopathia Striata with Cranial Stenosis, OSCS, respectively) implying a role for the WNT pathway in osteoclast function.

Mutations in genes described so far only account for approximately 70% of cases and the search continues for the genes responsible for the remainder. The field of osteopetrosis research has benefited from the many naturally occurring rodent models of the disease. Many genetic defects observed in rodents have not been observed in humans, and these form natural targets for future studies.

## Diagnosis

**Figure 5:**
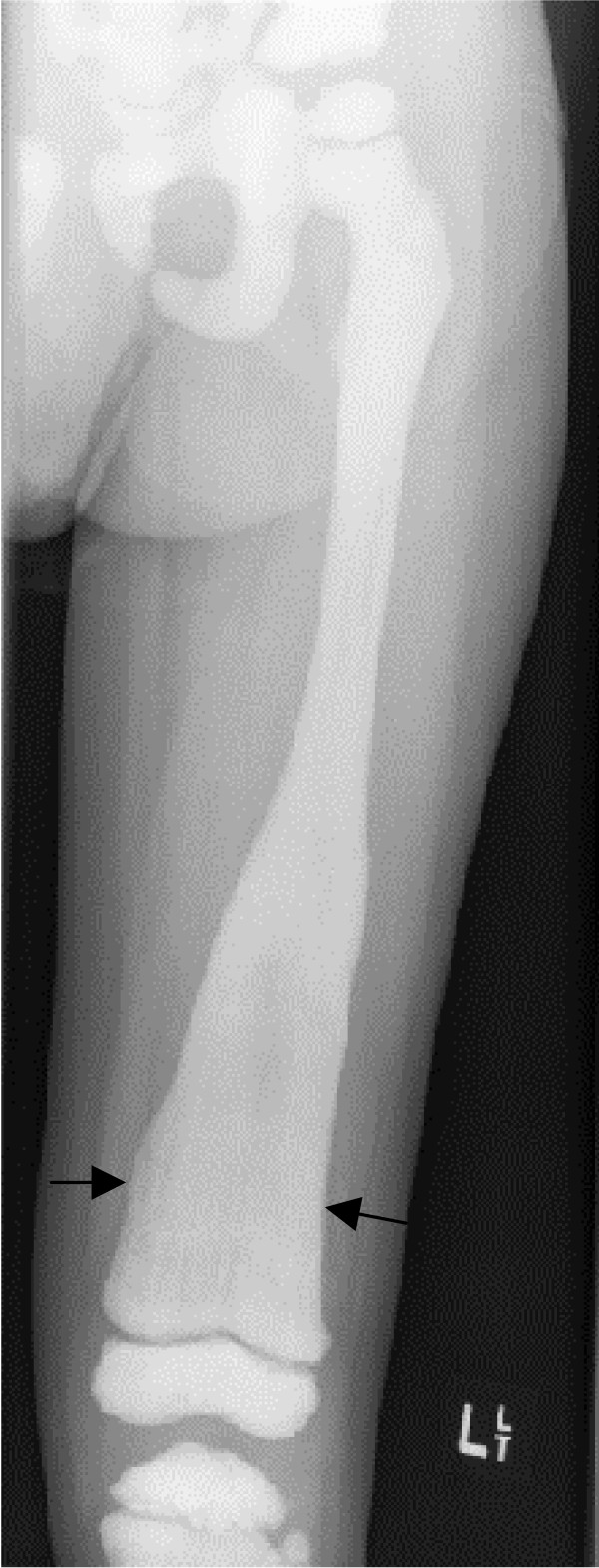
**ADO: Radiograph of left femur, age 4 years**. Note Erlenmeyer flask deformity of distal femur (arrows) and generalised increased bone density.

**Figure 6:**
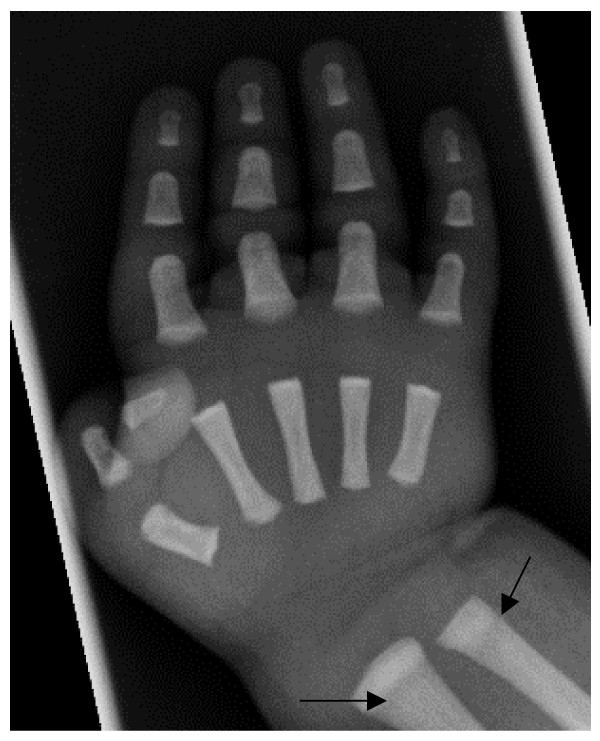
**Severe ARO: Right hand radiograph, age 2 weeks**. Note metaphyseal lucent bands in the distal ulna and radius (arrows) and short tubular bones.

The mainstay of diagnosis is clinical and largely depends on the radiographic appearance of the skeleton. The classic radiological features of osteopetrosis comprise:

• Diffuse sclerosis, affecting the skull, spine, pelvis and appendicular bones.

• Bone modelling defects at the metaphyses of long bones, such as funnel-like appearance ("Erlenmeyer flask" deformity) (Figure 5), and characteristic lucent bands (Figure 6).

• "Bone-in-bone" appearance particularly in the vertebrae and phalanges.

• Focal sclerosis of the skull base, pelvis and vertebral end plates – "sandwich" vertebrae (Figure 1) and "rugger-jersey" spine.

In the absence of typical radiographic findings, raised concentrations of the creatine kinase BB isoenzyme and tartrate resistant acid phosphatase (TRAP) can be helpful in making the diagnosis of ADO [63-65].

Age of onset, inheritance pattern and the presence of associated features, such as neurodegeneration, mental retardation, skin and immune system involvement, or renal tubular acidosis may point to particular subtypes of osteopetrosis. Bone biopsy can distinguish between osteoclast-poor and osteoclast-rich subtypes of ARO; however this is invasive and rarely performed.

Genetic testing is available either clinically or on a research basis for many of the genes implicated in osteopetrotic conditions. Genetic testing can be used to confirm the diagnosis and differentiate between different subtypes of osteopetrosis, providing additional information regarding prognosis, likely response to treatment and recurrence risks.

## Differential diagnosis

Primary sclerosing conditions of bone caused by osteoclast dysfunction need to be distinguished from the large number of conditions in which bone sclerosis can occur as a secondary phenomenon. Some alternative diagnoses to consider include fluorosis; beryllium, lead and bismuth poisoning; myelofibrosis; Paget's disease (sclerosing form); and malignancies (lymphoma, osteoblastic cancer metastases). Neonatal radiographs can be particularly difficult to interpret in the absence of additional multiorgan involvement, as the normal neonatal skeleton can appear denser than normal. However, in contrast to osteopetrosis, this appearance will improve over time.

Once the diagnosis of a primary osteopetrotic condition is made, it is important to distinguish between different subtypes as they have different response to treatment, prognosis and recurrence risks.

## Genetic counselling

Osteopetrosis is a trait that can be inherited in an autosomal dominant, autosomal recessive or X-linked manner, and genetic counselling will depend on the mode of inheritance in a particular family.

*Autosomal recessive*: the parents of the proband have a 1 in 4 (25%) risk of having further affected children in each pregnancy. 2/3 of unaffected siblings are expected to be carriers. Given the low incidence of osteopetrosis in the general population, the risk that the proband or their siblings would have affected children is low.

*Autosomal dominant*: the parents of the proband should be carefully evaluated for signs of osteopetrosis, including radiographic studies of the skeleton. Each child of an affected individual has a 1 in 2 (50%) risk of being affected. If the parents are unaffected, there may still be a low risk of further affected children due to gonadal mosaicism.

*X-linked recessive*: if the mother of the proband is a carrier, 50% of male pregnancies will be affected, and 50% of female pregnancies will be carriers. If the mother is not a carrier, there may still be a small risk of further affected offspring due to gonadal mosaicism.

## Antenatal diagnosis

Pre-implantation and prenatal diagnosis is theoretically possible in families, in whom the genetic mutation has been identified, thus allowing for reproductive decisions to be made. In families with severe ARO and unknown mutations, pre-natal diagnosis may be possible using radiographs [66]. If a family decides to continue with an affected pregnancy, haematopoietic stem cell transplantation (HSCT) before the age of 3 months can be planned with the aim of improving neurological outcomes.

## Management including treatment

At present, no effective medical treatment for osteopetrosis exists. Treatment is largely supportive and is aimed at providing multidisciplinary surveillance and symptomatic management of complications. Fractures and arthritis are common and require treatment by an experienced orthopaedic surgeon due to the brittleness of the bone, and the relatively frequent occurrence of secondary complications such as delayed union or non-union of fractures and osteomyelitis [67]. Hypocalcaemic seizures are treated with calcium and vitamin D supplementation, and bone marrow failure with red blood cell and platelet transfusions. Developmental delay and seizures in the setting of normal calcium levels may be indicative of neuropathic ARO and should prompt a formal neurological evaluation (including brain MRI, and EEG). Regular ophthalmologic surveillance including visual evoked potentials (VEPs) is important in detecting optic nerve atrophy. Surgical decompression of the optic nerve has been performed to prevent vision loss [68]. Dental problems such as delayed tooth eruption, ankylosis, abscesses, cysts and fistulas are common. Therefore, routine dental surveillance and maintenance of oral hygiene form an integral part of management and play an important role in preventing more severe complications such as osteomyelitis of the mandible.

Given the high associated morbidity and mortality, HSCT is reserved for the severe forms of ARO. HSCT using HLA-identical donors results in 73% 5-year disease-free survival [69]. Complications include rejection, delayed haematopoietic reconstitution, venous occlusive disease, pulmonary hypertension and hypercalcaemic crisis [70]. Furthermore HSCT does not necessarily reverse complications: retrospective report of HSCT in osteopetrosis [69] showed that only 7% of survivors experienced improvement in vision, whereas 69% had no further deterioration and 25% experienced further deterioration. HSCT had no effect on linear growth. Outcomes were better with earlier transplantation, particularly before the age of 3 months and of note, rescue of a malignant osteopetrosis mouse model by HSCT *in utero *has been demonstrated [71].

HSCT does not alter outcome in subtypes of osteopetrosis associated with primary rather than compressive neuropathy, such as autosomal recessive forms caused by *CLCN7 *and *OSTM1 *gene mutations. Other types of osteopetrosis that do not benefit from HSCT include those caused by absence rather than impaired function of osteoclasts (e.g. *RANKL *mutations) [36].

Interferon gamma 1b (IFNγ1b) treatment has been attempted in patients with osteopetrosis variants unlikely to respond to HSCT or as a bridge to transplantation. It has been reported to result in improvement in immune function, increase in bone resorption and increase in bone marrow space [72,73]. Stimulation of host osteoclasts with calcium restriction, calcitrol, steroids, parathyroid hormone and interferon has also been attempted [74,75].

## Prognosis

The severe infantile forms of osteopetrosis are associated with diminished life expectancy, with most untreated children dying in the first decade as a complication of bone marrow suppression. Life expectancy in the adult onset forms is normal.

## Unresolved questions

Despite recent advances in the understanding of the pathogenesis of osteopetrotic conditions, the genetic basis of approximately 30% of cases remains to be elucidated. The other major challenge in this group of conditions remains effective treatment of the severe recessive disorders and of complications such as optic and other cranial nerve compression. It is hoped that ongoing research into osteoclast physiology will result in novel therapeutic targets. For example, low levels of bone resorption are observed in even severely affected patients, pointing to the presence of multiple acidification mechanisms. The activation of alternative acidification mechanisms, including the Na+/H+ antiporter [65] have been proposed as potential therapeutic targets. The recent identification of RANKL-deficient patients has raised the possibility that this subgroup of patients would benefit from the administration of recombinant RANKL or from mesenchymal stem cell transplantation (MSCT)[76]. Feasibility studies of these approaches in animal studies are eagerly awaited.

## Competing interests

The authors declare that they have no competing interests.

## Authors' contributions

Both authors read and approved the final manuscript.
